# Immunogenicity of PvCyRPA, PvCelTOS and Pvs25 chimeric recombinant protein of *Plasmodium vivax* in murine model

**DOI:** 10.3389/fimmu.2024.1392043

**Published:** 2024-06-19

**Authors:** Ada da Silva Matos, Isabela Ferreira Soares, Rodrigo Nunes Rodrigues-da-Silva, Cinthia Magalhães Rodolphi, Letusa Albrecht, Rafael Amaral Donassolo, Cesar Lopez-Camacho, Ana Paula Dinis Ano Bom, Patrícia Cristina da Costa Neves, Fernando de Paiva Conte, Lilian Rose Pratt-Riccio, Cláudio Tadeu Daniel-Ribeiro, Paulo Renato Rivas Totino, Josué da Costa Lima-Junior

**Affiliations:** ^1^ Immunoparasitology Laboratory, Oswaldo Cruz Institute (IOC), Oswaldo Cruz Foundation (Fiocruz), Rio de Janeiro, Brazil; ^2^ Hantaviruses and Rickettsioses Laboratory, IOC, Fiocruz, Rio de Janeiro, Brazil; ^3^ Parasitology Research Center, Federal University of Juiz de Fora (UFJF), Juiz de Fora, Brazil; ^4^ Apicomplexa Research Laboratory, Carlos Chagas Institute, Curitiba, Brazil; ^5^ Nuffield Department of Medicine, The Jenner Institute, University of Oxford, Oxford, United Kingdom; ^6^ Immunological Technology Laboratory, Immunobiological Technology Institute (Bio-Manguinhos/Fiocruz), Rio de Janeiro, Brazil; ^7^ Eukaryotic Pilot Laboratory, Immunobiological Technology Institute (Bio-Manguinhos/Fiocruz), Rio de Janeiro, Brazil; ^8^ Malaria Research Laboratory, IOC, Fiocruz, Rio de Janeiro, Brazil

**Keywords:** plasmodium vivax, multistage chimeric protein, immunization, BALB/c, in silico simulation, vaccine, immunogenicity

## Abstract

In the Americas, *P. vivax* is the predominant causative species of malaria, a debilitating and economically significant disease. Due to the complexity of the malaria parasite life cycle, a vaccine formulation with multiple antigens expressed in various parasite stages may represent an effective approach. Based on this, we previously designed and constructed a chimeric recombinant protein, PvRMC-1, composed by PvCyRPA, PvCelTOS, and Pvs25 epitopes. This chimeric protein was strongly recognized by naturally acquired antibodies from exposed population in the Brazilian Amazon. However, there was no investigation about the induced immune response of PvRMC-1. Therefore, in this work, we evaluated the immunogenicity of this chimeric antigen formulated in three distinct adjuvants: Stimune, AddaVax or Aluminum hydroxide (Al(OH)3) in BALB/c mice. Our results suggested that the chimeric protein PvRMC-1 were capable to generate humoral and cellular responses across all three formulations. Antibodies recognized full-length PvRMC-1 and linear B-cell epitopes from PvCyRPA, PvCelTOS, and Pvs25 individually. Moreover, mice’s splenocytes were activated, producing IFN-γ in response to PvCelTOS and PvCyRPA peptide epitopes, affirming T-cell epitopes in the antigen. While aluminum hydroxide showed notable cellular response, Stimune and Addavax induced a more comprehensive immune response, encompassing both cellular and humoral components. Thus, our findings indicate that PvRMC-1 would be a promising multistage vaccine candidate that could advance to further preclinical studies.

## 1 Introduction

Malaria continues to pose a substantial global public health threat, with the WHO estimating 249 million cases in 2022, and more than 2 million deaths between 2019 and 2022 (1). While *Plasmodium falciparum* is the predominant malaria parasite in Africa and certain parts of Asia, *P. vivax* malaria is the most widely distributed species, particularly prevalent in Central and South America, where it constituted almost 71.5% of cases in 2021 (2, 3). In Brazil, 128,984 confirmed malaria cases were recorded in 2022, with 84% attributed to *P. vivax*. Notably, severe and life-threatening vivax malaria is no longer considered a rare event (3). The licensed malaria vaccine, RTS, S, targets *P. falciparum* sporozoites but lacks cross-protection against *P. vivax*, emphasizing the need for an effective vaccine targeting *P. vivax* to control the resurgence and progress towards malaria elimination outside Africa.

In the field of malaria vaccine development, strategies are focused around the *Plasmodium* biological cycle, aiming to stimulate stage-specific immune responses (4, 5). These approaches include pre-erythrocytic strategies to neutralize sporozoites and prevent hepatocyte invasion (6, 7), erythrocytic vaccines to inhibit merozoite invasion and multiplication (8–10), and transmission-blocking vaccines against sexual stages to impede mosquito infectivity (11, 12). Given the complexity of the *Plasmodium* spp. life cycle, the optimal vaccine design should address all stages (13, 14). The prevailing belief is that a vaccine capable of inducing immunogenic responses across multiple stages of parasite development would provide superior protection.

In this context, three *P. vivax* proteins involved in different phases of the biological cycle were explored recently by our group for a multistage vaccine candidate composed of epitopes from PvCelTOS (Cell-Traversal Protein For Ookinetes And Sporozoites), PvCyRPA (Cysteine-Rich Protective Antigen) and Pvs25 (Ookinete Surface Protein) (15–18). Briefly, CelTOS is vital for the malaria parasite’s hepatocyte traversal, considered an attractive vaccine candidate due to its high conservation among *Plasmodium* species (19, 20). PfCelTOS peptides stimulate peripheral blood mononuclear cells (PBMCs), correlating with enhanced protection (21). Immunization with PfCelTOS induces sterile protection in mice against *P. berghei* sporozoites (22). PvCelTOS, explored in our previous studies in Brazilian endemic areas, exhibits natural immunogenicity and conservation with worldwide isolates (15, 16), making it a valuable antigen for a *P. vivax* vaccine. Concerning the erythrocytic stage, CyRPA (cysteine-rich protective antigen) plays a key role in merozoite invasion (23). Despite basigin not being essential for *P. vivax* invasion, CyRPA remains crucial, and antibody responses to it correlate strongly with protection (24). PvCyRPA, despite moderate sequence variation, retains significant antibody targets (17). Regarding transmission, Pvs25 is expressed in mosquito stages with minimal genetic variation (18). Mouse antisera to Pvs25 prevent oocyst development, and antibody levels correlate with transmission-blocking activity in vaccine trials (25–27), establishing Pvs25 as a promising transmission-blocking vaccine candidate. Lastly, all three promising candidate proteins presented predicted and/or confirmed T and B cell epitopes, which represented compelling targets for comprehensive malaria vaccine development (15–18).

Recently, we designed and expressed a *P. vivax* recombinant modular chimeric protein (PvRMC-1) composed of the main antigenic regions of these vaccine candidates (13). The protein was successfully expressed and the predicted structure retained the stage-specific epitopes. Moreover, in addition to the antigenicity confirmation by IgG and IgM antibody recognition, the PvRMC-1 seroprevalence in a population naturally exposed to malaria revealed a high frequency of total antibody responders, predominantly displaying cytophilic IgG1. Indeed recent infected individuals presented higher antibody response, suggesting the potential of this antigen as a successful immunogen (13). However, conclusive insights into the immunogenicity of PvRMC-1 will not be achieved without immunization studies in animal models. In this context, non-humanized mouse models serve as vital tools for evaluating malaria vaccine candidates targeting *P. vivax*. It facilitates the assessment of vaccine formulations’ immunogenicity, aiding in the selection of promising candidates for further investigation. By exposing mice to recombinant antigens formulated with various adjuvants, the immune response elicited against *P. vivax* antigens can be evaluated. While such studies provide valuable insights into recombinant constructs and immunogenicity, they are often a precursor to more complex evaluations in established non-human primate models, aiming to bridge the translational gap towards human clinical trials.

Therefore, in this study we aimed to evaluate PvRMC-1 as an immunogen in different adjuvant formulations, using BALB/c mice as a model. We aimed to investigate the cellular and humoral immune response against PvRMC-1 and its protein epitopes.

## 2 Materials and methods

### 2.1 Recombinant protein

PvRMC-1 design, topology, and successful expression in *Escherichia coli* were described earlier (13). Briefly, the chimeric recombinant protein was designed with four T-cell epitopes and two B-cell epitopes from PvCelTOS, two linear B-cell epitopes from Pvs25, and a 255 amino acid sequence containing both B- and T-cell epitopes of PvCyRPA, encompassing the entire protein. The protein was subsequently expressed in *E. coli* and purified to 95% purity.

### 2.2 Immunization of mice with PvRMC-1

**Figure 1:**
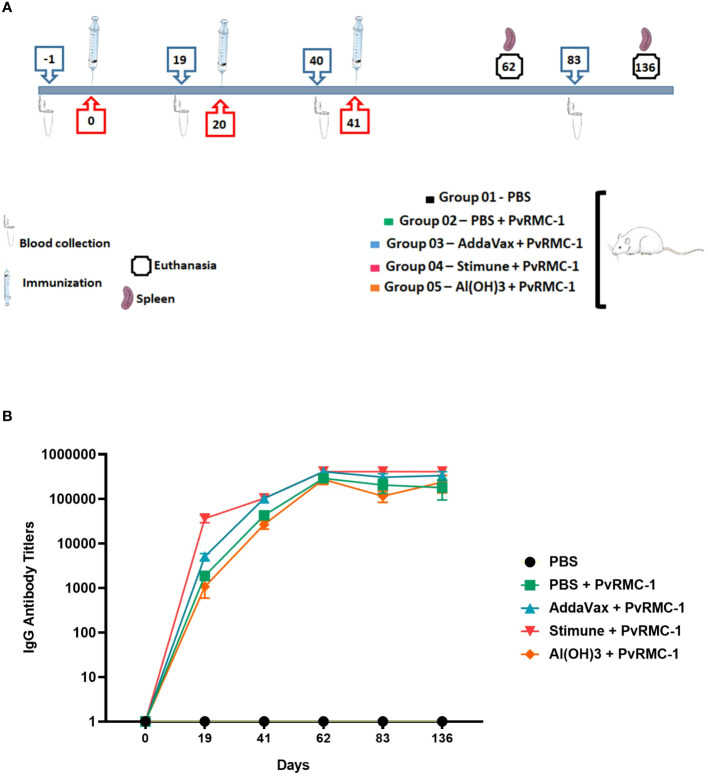
Kinetics with immunization, blood collection, and euthanasia phases. **(A)** BALB/c mice were separated into groups and described above according to the numbering, maintained throughout the study. Each symbol is identified below with the captions. **(B)** BALB/c mice (n = 8 per group) were immunized intramuscularly with PvRMC-1 formulated with three adjuvants: Al(OH)3, AddaVax, and Stimune. PBS was used as a control. ELISA was performed to calculate antibody titers at all time points. Throughout the follow-up time, a continuous increase in response was observed, even in the most diluted serum samples. The group immunized with the adjuvant Stimune exhibited the most prominent response, followed by AddaVax, Antigen + PBS, and lastly Al(OH)3.

Groups of 6–8 weeks BALB/c mice were obtained from the Institute of Science and Technologies in Biomodels (ICTB)/FIOCRUZ and animal immunizations were performed in LAEAN, the Animal Experimentation Laboratory at Bio-Manguinhos/FIOCRUZ. Five groups were organized, each containing eight animals, with half composed of males and the other half of females. The immunization was performed three times via subcutaneous injection in the abdominal flank region, with an interval of about 21 days between each dose, which was comprised of 50 µg recombinant protein in 100 µl PBS or adjuvant: Stimune (Prionics, Schlieren-Zurich, Switzerland), AddaVax (InvivoGen, San Diego, CA, USA) or Al(OH)3 (Sigma-Aldrich, Saint Louis, MO, USA). During the kinetic study, blood samples (40µl/mice) were collected via the retro-orbital route on days -1, 19, 40, 62, and 83, so that the obtained serum was used for ELISA assays to evaluate the antibody response. For the evaluation of cellular response using ELISPOT assay, half animals were sacrificed at day 62 and the other half at day 136, and spleens were used. Total IgG was detected by Enzyme-linked immunosorbent assay (ELISA) in all time points and IgG subclasses were evaluated on days 19 and 62 (**Figure 1**). All these procedures were done in independent experiments and following the animal welfare protocols established by Bio-Manguinhos (CEUA: LW-13/16).

### 2.3 Antibody assays

The presence and levels of specific antibodies against the synthetic peptides in the sera of mice were evaluated by ELISA. Briefly, 96-microwell plates (Nunc, Rochester, NY, USA) were coated with 100 ng/ml of PvRMC-1 and 100ul have been added to each well. After overnight incubation at 4 °C, the plates were washed with PBS and blocked with PBS-0.05% Tween 20 containing 5% nonfat dry milk (PBS-Tween 5%) for 1 h at 37°C. Individual serum samples at two-fold serial dilutions in PBS-Tween-2.5% skim milk (PBS-Tween-M) were added to duplicate wells, and the plates were incubated at 37°C for 1 h. After three washes with PBS-Tween, bonded antibodies were detected with peroxidase-conjugated goat anti-mouse IgG (1:4000) (Southern Biotech, Birmingham, AL) followed by o-phenylenediamine and hydrogen peroxide. The absorbance was read at 490 nm using an ELISA reader (Spectramax 250, Molecular Devices, Sunnyvale, CA). The end-point titers in the mice sera were determined as the highest dilution at which immunized mice sera had optical density (OD) value three times higher than sera from control mice (the OD values in the control mice were about 0.058 for IgG; 0.089 and 0.068 for subclasses in day 62 and 136, respectively). The determination of the IgG subclass profile was also performed as described above, except that the secondary antibodies used were goat anti-mouse monoclonal antibodies specific for mouse IgG1, IgG2a, IgG2b, or IgG3 (1:4000) (Southern Biotech, Birmingham, AL).

### 2.4 Peptide synthesis

The PvCelTOS and PvCyRPA B and T-cells and Pvs25 B-cells chosen to compound the PvRMC-1 were based on the best results from *in silico* prediction, experimental confirmation and genetic diversity (15–18) and finally synthesized using fluorenyl methoxycarbonyl (F-moc) solid-phase chemistry by GenOne Biotechnologies, RJ, Brazil (**Table 1**). All peptides showed a purity of >90% determined by HPLC (high-performance liquid chromatography).

**Table 1:** T and B-cell peptides synthesized. Synthetic Peptides antigens used in immunoassays (ELISA and ELISpot) by target protein epitope, position, and sequence.

Target	Peptide ID	Position	Sequence
T-cell epitope (PvCyRPA)	T1	PvCyRPA T49-E63	TEIHVLVQKKINSTWE
	T2	PvCyRPA L54-T68	LVQKKINSTWETQTT
	T3	PvCyRPA R102-T116	REGTICKRWNSVTGT
	T4	PvCyRPA D167-T181	DNFISCVASEDKGRT
	T5	PvCyRPA Y216-Y230	YSRISTNNTARGGNY
	T6	PvCyRPA T221-L235	TNNTARGGNYMTCTL
	T7	PvCyRPA T232-K246	TCTLDVTNEGKKEYK
	T8	PvCyRPA Y287-Q301	YYTEQNAIVVKPKVQ
	T9	PvCyRPA Q306-K320	QNDDLNGCYGGSFVKLDESK
	T10	PvCyRPA Y340-D354	YGVQNIHTLYYTRYD
B-cell epitope (PvCyRPA)	B1	PvCyRPA I58-L68	INSTWETQTTL
	B2	PvCyRPA Y96-I106	YKQRSKREGTI
	B3	PvCyRPA N111-E128	NSVTGTIYQKEDVQIDKE
	B4	PvCyRPA S158-F169	SYEYKTANKDNF
	B5	PvCyRPA R218-R226	RISTNNTAR
	B6	PvCyRPA T234-C249	TLDVTNEGKKEYKFKC
	B7	PvCyRPA T289-G307	TEQNAIVVKPKVQNDDLNG
T-cell epitope (PvCelTOS)	T1	PvCelTOS I133-G147	IKPPRVSEDAYFLLG
	T2	PvCelTOS P139-V153	PRVSEDAYFLLGPVV
	T3	PvCelTOS 145–159	DAYFLLGPVVKTLFN
	T4	PvCelTOS 151–165	GPVVKTLFNKVEDVL
	T5	PvCelTOS 157–171	LFNKVEDVLHKPIPD
	T6	PvCelTOS 163–177	DVLHKPIPDTIWEYE
	T7	PvCelTOS 169–183	IPDTIWEYESKGSLE
	T8	PvCelTOS 176–189	YESKGSLEEEEAED
	T9	PvCelTOS 181–195	LEEEEAEDEFSDELL
	T10	PvCelTOS 189–196	EFSDELLD
B-cell epitope (PvCelTOS	B1	PvCelTOS P127-V153	PTEKIVASTIKPPRVSEDAYFLLGPVVKTLFNKVEDV
	B2	PvCelTOS L181-D196	LHKPIPDTIWEYESKGSLEEEEAEDEFSDELLD
B-cell epitope (Pvs25)	B1	Pvs25 L53-A72	LSENTCEEKNECKKETLGKA
	B2	Pvs25 I139-A158	IGKVPNPEDEKKCTKTGETA

### 2.5 ELISpot assays

An ELISpot kit (Mabtech, Nacka Strand, Sweden) was used. Briefly, cell cultures were carried out in duplicate in pre-coated IFN- γ nitrocellulose 96 well plates. Plates were blocked with RPMI medium (Gibco; Thermo Fisher Scientific, Inc., Waltham, MA, USA) containing 10% fetal calf serum for 30 minutes and 2.5×10^5^ cells were added to the ELISPOT plates in the presence of medium alone to act as a control or with 10 μg/ml of different pool combinations of PvCyRPA or PvCelTOS derived peptides (**Table 2**), and Concanavalin A was used as positive control. To determine IFN-γ secretion, cells were stimulated for 24 h at 37°C, 5% CO2 under sterile conditions. After stimulation, plates were washed four times with PBS 1X and incubated with biotin-anti-human IFN-γ Clone 7-B6–1 (MabTech) diluted in PBS 1X containing 0.05% of fetal calf serum for 2 h at 37°C. The plates were washed four times with PBS 1X and incubated with streptavidin-alkaline phosphatase (MabTech) in PBS 0.05% for 1 h at 37°C. The plates were washed four times with PBS 1X before development with 1-step NBT/BCIP. Development was stopped by the addition of distilled water. IFN-γ secreting cells appeared as purple spots and were counted with an Immunospot reader (Cellular Technology Ltd, Cleveland, OH) using the Immunospot Software. The responses were accessed by the mean number of SFC in peptide-stimulated wells minus the mean number of SFC in control wells with medium alone from the same mice. Results were expressed as spot-forming cells (SFC) per million PBMCs.

**Table 2:** Composition of peptide pools. Composition of T and B cell epitope pools for PvCyRPA and PvCelTOS and B cells for Pvs25.

Pool ID	Target	Composition
T-cell epitope Pool 1	PvCyRPA	T3, T4, T5 and T6
T-cell epitope Pool 2		T1, T3, T5 and T9
T-cell epitope Megapool		T1, T2, T3, T4, T5, T6, T7, T8, T9 and T10
T-cell epitope Pool 1	PvCelTOS	T1, T2, T3, T4 and T5
T-cell epitope Pool 2		T6, T7, T8, T9 and T10
B-cell epitopes Pool	PvCelTOS	B1 and B2
	PvCyRPA	B1, B2, B3, B3, B5, B6 and B7
	Pvs25	B1 and B2

### 2.6 Recognition of B-cell epitopes (Peptide Elisa)

To confirm the recognition of B-cell epitopes that compose PvRMC-1, ELISA was performed. MaxiSorp 96-well plates (Nunc, Rochester, NY, USA) were coated with PBS containing 50 µg/ml of each pool of PvCeltos peptides, PvCyRPA peptides, and Pvs25 peptides, followed by incubation at 37°C in the humid incubator. The plates were washed and blocked with 4%BSA for 1h and 30 min at 37°C, in the humid incubator. After this, the individual serum samples were diluted 1:100 in PBS-Tween containing 2%BSA in duplicate wells. After 2 h at 37°C in the humid incubator and three washings with PBS-Tween, bound antibodies were detected with peroxidase-conjugated goat antihuman IgG (1:2000) (Sigma, St. Louis) and followed by addition of o-phenylenediamine and hydrogen peroxide. Optical density was identified at 490 nm using a SpectraMax 250 ELISA reader (Molecular Devices, Sunnyvale, CA, USA). As made for recombinant protein, the results for each peptide were expressed based on the average optical density (OD) of each animal.

### 2.7 Statistical analysis

All statistical analyses were carried out using Prism 9.0 for Windows (GraphPad Software, Inc., Software, Inc., San Diego, CA, USA). Categorical variables’ statistical differences between the two defined groups were assessed using the Fisher exact test, while differences in continuous variables were determined using the Mann-Whitney U test. Statistically significant results were defined as p-values of ≤0.05. A multiple comparisons test was used to compare ODs of IgG against recombinant PvRMC-1 between the studied groups. Heatmap was performed with Matplotlib Library using Python language (28).

### 2.8 *In silico* simulation of immune response profile in humans

The immune profiles of the designed vaccine were assessed using the C-ImmSim online server (https://kraken.iac.rm.cnr.it/C-IMMSIM), which mimics the natural immune environment and stimulates the immune response in humans, evaluating the immunogenicity testing and the determination of the immune response profile. The C-ImmSim server uses machine-learning techniques to predict immune responses based on three compartments: lymph nodes, thymus, and bone marrow (29). The sequence of the chimeric protein was used as an input. Three injection doses considering 1000 immunogen proteins each, were performed with 21 days of interval (1, 21, and 42) and the simulation was conducted for 180 days. Simulation steps were adjusted to 500 and other parameters were kept at the default.

## 3 Results

### 3.1 PvRMC-1 is immunogenic in different adjuvant formulations

Initially, we immunized five groups of BALB/c mice, each consisting of 8 animals (**Figure 1A**). To access the kinetics of IgG antibody titers blood samples were collected from the animals at six time points. We observed a robust B-cell-mediated response in all time points, requiring further dilution during the ELISA assays to explore the magnitude of these responses. The PvRMC-1 protein was immunogenic in any of the formulations, and the group immunized with PBS alone showed no response at any of the assessed time points. However, we noted a difference in the response kinetics when comparing the different adjuvants. Mice immunized with Stimune exhibited the highest antibody titers immediately after the first immunization (mean=36,266.7), while the other formulations showed lower titers on day 19, with the PvRMC-1 and PBS (mean=1,866.67) or Al(OH)3 (mean=1,066.67) groups presenting lower titers and those immunized with PvRMC and Addavax presenting intermediate levels (mean=5,066.67). Nevertheless, after the third immunization, although the Stimune and Addavax groups showed higher titers (mean= 409,600 and 332,800, respectively), all formulations were capable of generating high antibody titers up to 136 days after the initial immunization (mean=236,800, for AL(OH)3). Finally, we noted that after the third immunization, only the PvRMC-1 and PBS-immunized group began to show a trend of declining titers after day 62 (mean=290,133) until day 136 (mean=179,200). The other groups did maintain high titers regardless of the adjuvant used (**Figure 1B**).

### 3.2 IgG1 and IgG2 are the predominant isotypes against PvRMC-1

**Figure 2:**
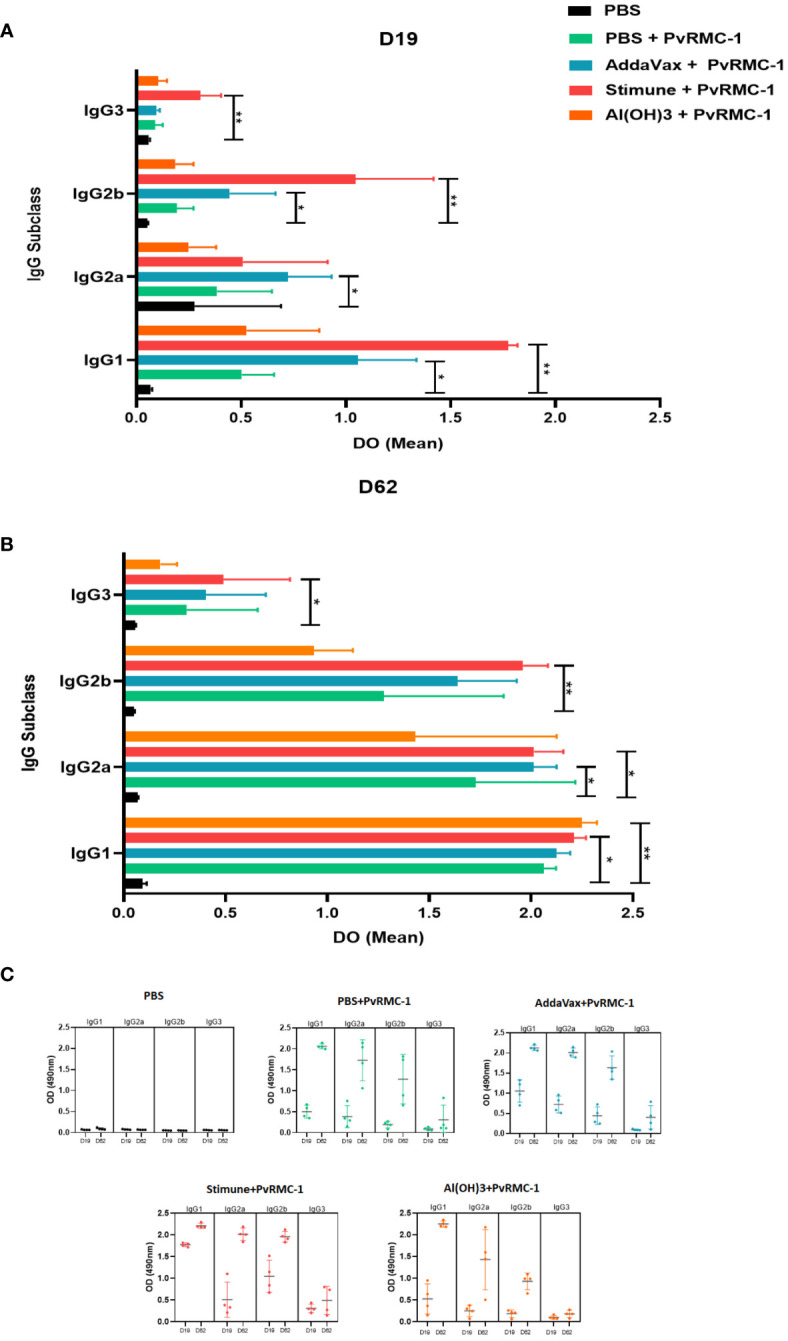
IgG isotype mean Optical Density (OD) magnitude compared to the control group. **(A)** Enzyme-linked immunosorbent Assay (ELISA) was performed to calculate antibody titers in Day 19 sera. The mean OD for each subclass was calculated and compared inside each IgG subclass group. IgG1 and IgG3 Stimune were higher than the control group. The same response was observed for IgG2a and IgG2b of its adjuvant. IgG2b AddaVax performed the same scenario. **(B)** On Day 62, Stimune IgG1, IgG2a, and IgG3 continued higher than the control, as observed for IgG2a of AddaVax In this point of kinects, IgG1 Al(OH)3 was also higher than the control group. The P values were calculated by two-way ANOVA with the Kruskal-Wallis test (* P ≤ 0.05 ** P ≤ 0.01). **(C)** The complete scenario of the mean OD values for IgG subclasses for each immunized group on the two ELISA assay days. In D19 the variations of OD means and Standard Deviation (SD) values for the control group PBS (IgG1 = 0.067 SD = 0.007; IgG2a =0.075; DP = 0.008; IgG2b = 0.051; DP = 0.004; IgG3 = 0.059; DP = 0.005, antigen alone (IgG1 = 0.503; SD = 0.153; IgG2a = 0.385; DP = 0.261; IgG2b = 0.193. DP = 0.079; IgG3 = 0.089; DP = 0.035), AddaVax (IgG1 = 1.058; SD = 0.278; IgG2a = 0.723; DP = 0.208; IgG2b = 0.449; DP = 0.219; IgG3 = 0.096; DP = 0.015), Stimune (IgG1 = 1.775; SD = 0.004; IgG2a = 0.508; DP = 0.404; IgG2b = 1.046; DP = 0.372; IgG3 = 0.306; DP = 0.097), and Al(OH)3 (IgG1 = 0.526; DP = 0.346; IgG2 = 0.249; DP = 0.131; IgG2b = 0.186; DP = 0.086; IgG3 = 0.103; DP =0.042) are presented, as well as for D62 for PBS (IgG1 = 0.091; SD = 0.021; IgG2a = 0.067; DP = 0.004; IgG2b = 0.050; DP = 0.003; IgG3 = 0.057; DP = 0.003) antigen alone (IgG1 = 2.062; SD = 0.060; IgG2a = 1.727; DP = 0.004; IgG2b = 1.276; DP = 0.589; IgG3 = 0.309; DP = 0.348), AddaVax (IgG1 = 2.126; SD = 0.066; IgG2a = 2.011; DP = 0.114; IgG2b = 1.639; DP = 0.289; IgG3 = 0.403; SD = 0.294), Stimune (IgG1 = 2.211; SD = 0.058; IgG2a = 2.015; DP = 0.114; IgG2b = 1.960; DP= 0.123; IgG3 = 0.489; DP = 0.326) and Al(OH)3 (IgG1 = 2.251; DP = 0.071; IgG2a = 1.432; DP = 0.692; IgG2b = 0.936; DP = 0.192; IgG3 = 0.179; DP = 0.082).

For the IgG subclasses, we evaluated whether there was a significant difference between the OD of the control groups immunized only with PBS about each of the tested adjuvants. We observed on Day 19 of kinetics a significant difference for IgG1 response in the group immunized with the adjuvant Stimune, compared to PBS control (p= 0.0022), a result that was also found for AddaVax group in a lower magnitude (p= 0.0497). For the IgG2a subclass, we observed that the group immunized with AddaVax had a significant difference (p= 0.010). For IgG2b, a difference was found between the groups immunized with AddaVax *vs* PBS and Stimune *vs* PBS (p= 0.0497 and p= 0.0017, respectively). Finally, for IgG3, a significant difference was observed only in the group immunized with Stimune (p= 0.0027) (**Figure 2A**).

On day 62 of our kinetic analysis, we observed statistical differences within the IgG1 subclass between Stimune and its PBS control (p = 0.0341), as well as for Al(OH)3 (p = 0.0053). For IgG2a, there was a difference compared to the group immunized with Stimune and AddaVax (p = 0.0496 for both). Regarding IgG2b and IgG3, only Stimune exhibited statistical differences (p = 0.0027 and p = 0.0341, respectively) (**Figure 2B**). In a complementary approach, we examined the variation of OD in all studied groups on both tested immunization days for subclasses (**Figure 2C**). We observed that, in a general scenario, after the third immunization, the average OD increased, with particular emphasis on IgG1 and IgG2a.

In addition, we assessed whether there was a difference in the magnitude of OD values among the subclasses, considering the evaluation within each of the tested adjuvant groups in our study. Consequently, on Day 19, we found a statistical difference between the Stimune group in all IgG subclasses. IgG1 has the highest OD compared to IgG2a (p= 0.028), IgG2b (p= 0.028) and IgG3 (p= 0.008). The IgG1 subclass from the group immunized only with PvRMC-1 also showed a statistical difference compared to the IgG2b (p= 0.028) and IgG3 (p= 0.010) subclasses; IgG1 from the group immunized with AddaVax adjuvant also showed a difference in OD means compared to IgG3 (p= 0.005). Applying the same type of analysis on day 62 of the kinetic study, we observed a significant statistical difference between IgG1 of all studied groups and IgG3, with p= 0.0084 for the antigen, p= 0.005 for AddaVax, p= 0.003 for Stimune, and p= 0.002 for Al(OH)3.

### 3.3 Antibodies from PvRMC-1 Immunized mice recognized B-cell epitopes from PvCyRPA, PvCelTOS and Pvs25

Complementary to assess the specific recognition of PvRMC-1, we conducted an ELISA assay with B cell epitopes from PvCelTOS, PvCyRPA, and Pvs25. For PvCelTOS and PvCyRPA we used peptide pools to encompass most of the B cell epitopes in the chimeric structure, along with two peptides from Pvs25.

**Figure 3:**
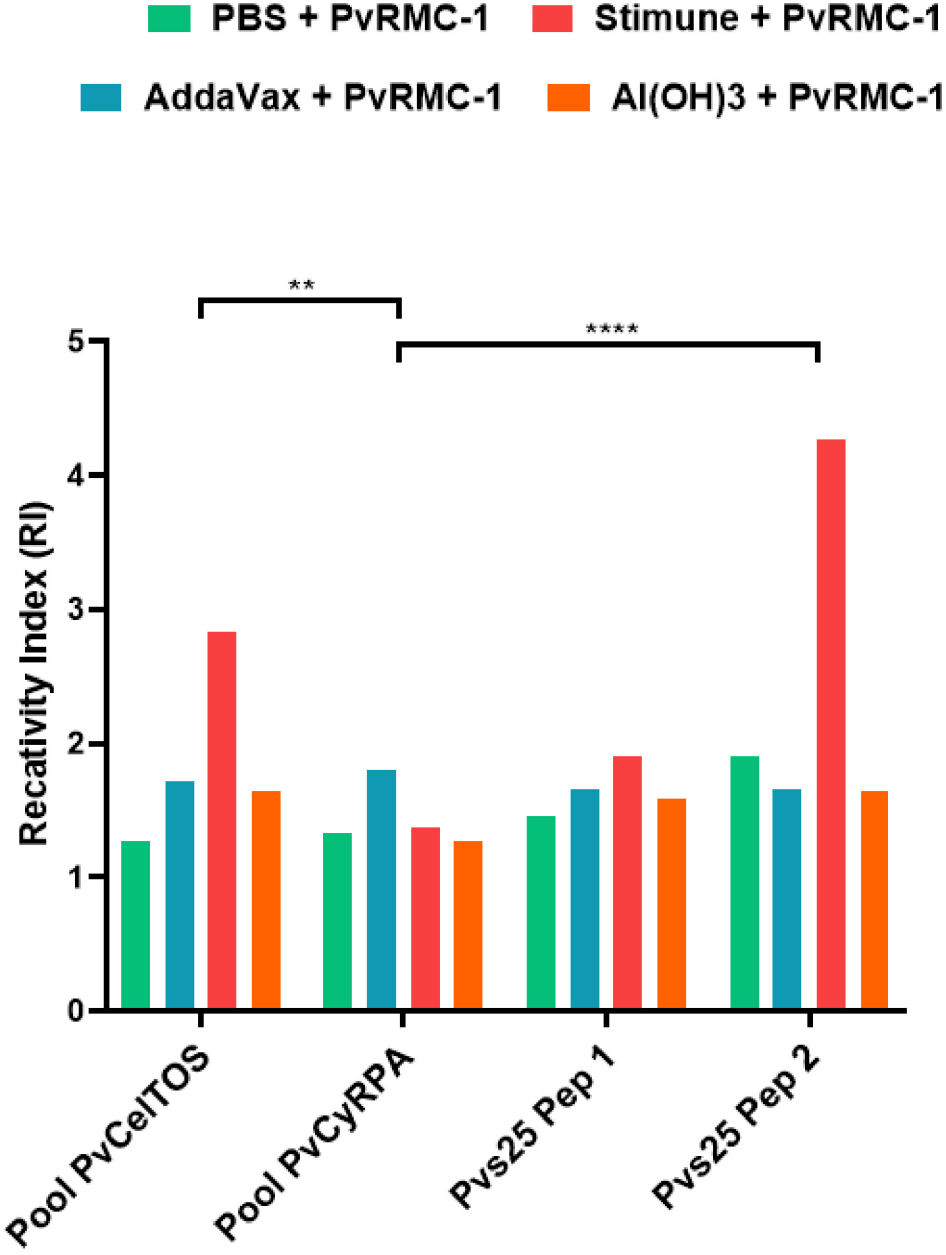
The magnitude of B-cell peptides OD for each group immunized. Enzyme-linked immunosorbent assay (ELISA) was performed with sera isolated from BALB/c immunized in D62. To calculate the cut-off value, we used the mean OD adjuvanted-group/OD mean PBS. The numbers on the Reactivity Index (RI) bar represent how much each group was higher when compared to the control group (PBS). (**p<0.01 and ****p<0.0001).

Firstly, we observed that the humoral immune response induced against PvRMC-1 generated antibodies capable of recognizing the epitopes of the different proteins, in any of the formulations used, (**Figure 3**). Confirming the successful representation of the chimeric protein about the selected epitopes and the potential targeting of different stages of the parasite’s life cycle. Thus, when evaluating each of the immunized groups individually, we found that the group adjuvanted with Stimune exhibited higher reactivity indexes for almost all tested peptides and linear epitopes from PvCelTOS and Pvs25 than from peptides of PvCyRPA (PvCelTOS vs. PvCyRPA, p= 0.001 and Pvs25 Peptide 2 vs. PvCyRPA, p<0.0001). The control group (only PBS) presented no response to individual epitopes.

### 3.4 PvCelTOS and PvCyRPA T-cell epitopes stimulate IFN-γ production in splenocytes of immunized mice

**Figure 4:**
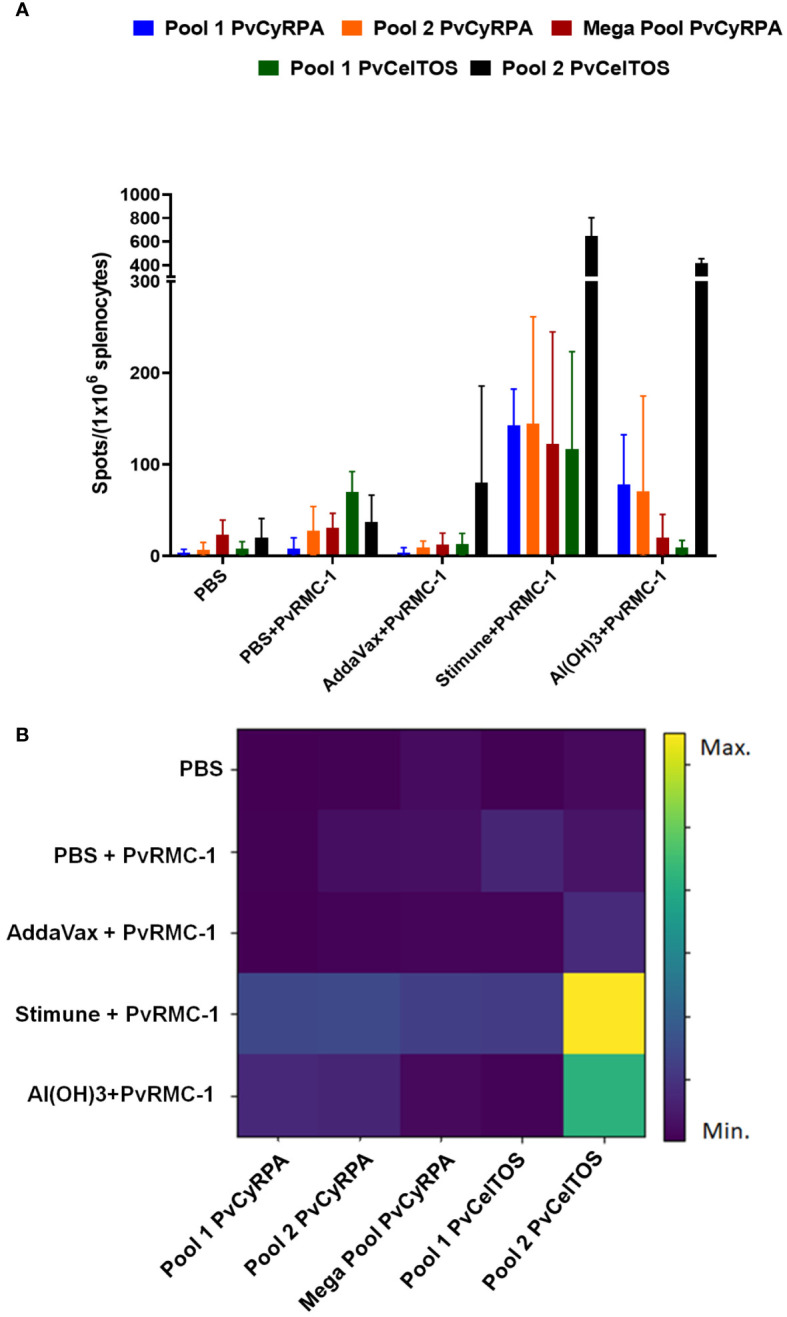
Cellular immune response scenario. Enzyme-linked immunosorbent spot (ELISpot) was performed in Day 62 splenocyte cells to evaluate after the complete vaccine scheme. **(A)** Number of spots stimulated for each T-cell peptide tested in all immunized groups. **(B)** Heatmap of spots mean for each immunized group. The closer to the yellow shade on the intensity bar, the higher the mean found. Stimune and Al(OH)3 showed the highest intensities, especially for Pool 2 of PvCelTOS. AddaVax demonstrated a lower intensity, often close to the blue shade observed for the control group (PBS).

Once observed that B-cell epitopes were broadly recognized by immunized animals, we also explored the cellular immune responses evaluating the effect of T-cell epitope stimulation on mice splenocytes. To determine the number of IFN-γ Spots Forming Cells (SFC) induced by immunization with PvRMC-1 and different adjuvants, we used the ELISPOT. Splenocytes derived from mice immunized with the different PvRMC-1 formulations and control group were collected 3 weeks after the third immunization (day 63) and were stimulated *ex vivo* using T-cell peptide pools from PvCyRPA and PvCelTOS (vertebrate host antigens) that achieved the highest immunogenicity scores in our previous works (14). PvCelTOS Pool 2 exhibited a higher number of spots, and all epitopes were more immunogenic in groups immunized with Stimune and Al(OH)3, especially for PvCelTOS Pool 2 (**Figure 4A**). However, the groups immunized with PvRMC-1 in PBS or AddaVax presented a low capacity for inducing an immune response when splenocytes were stimulated. Quantifying the raw number of spots, no statistical difference was observed within each of the studied groups. However, when evaluating the overall response of each utilized pool, we found that PvCelTOS Pool 2 is significantly more expressive than Pool 1, Pool 2, and Megapool of PvCyRPA and PvCelTOS Pool 1 (p<0.0001) (**Figure 4B**). All cells stimulated with Concanavalin A have >5000 IFN-γ-secreting cells per million cells.

### 3.5 Simulation of the immunogenicity of PvRMC-1 in human models

**Figure 5:**
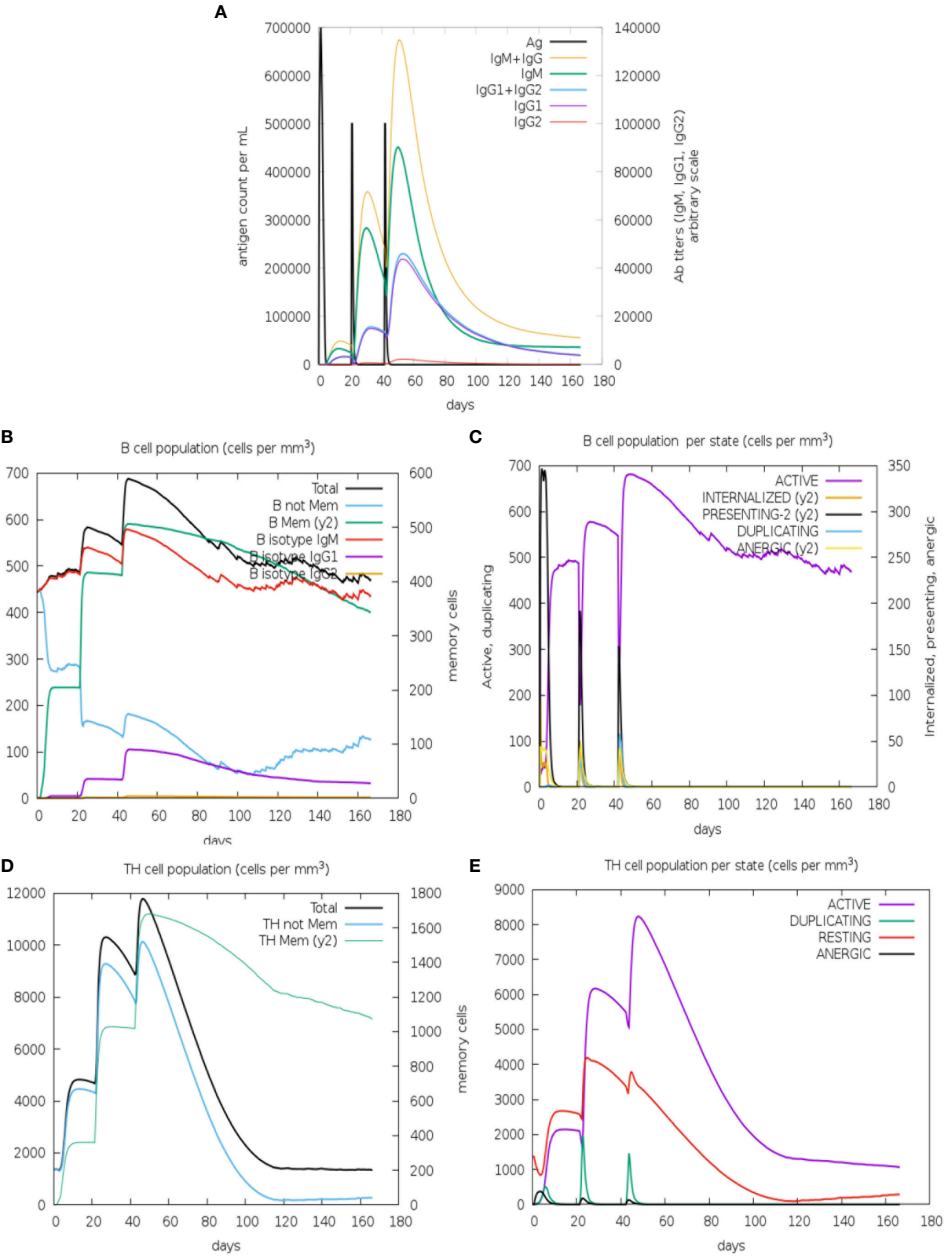
Immune simulation prediction: **(A)** Antigen and immunoglobulins predicted dynamics, with high antibody titers reached only after the third immunization (IgM+IgG). **(B)** B lymphocytes: total count and sub-divided. Memory B cells were predicted to reach the peak after the third dose and non-memory B-Cells decay along the kinetics **(C)** B lymphocyte population per entity-state also predict the active B-cell being maintained after 160 days. The CD4 T-helper lymphocytes count also shows a high peak of memory T-cells **(D)** and active T-cells **(E)** and after the third dose. The plot shows total and memory counts. D0/D20/D40 = first, second, and thirst doses.

To complement our work, we evaluated the immunogenicity response of PvRMC-1 in human models through a simulation in a three-dose scheme using artificial intelligence (**Figure 5**). The simulations were performed using only the action of the chimeric protein alone, without the use of any adjuvant, allowing us to evaluate the potential of the response generated in isolation. Parameters such as titration, separation into populations, and the state of B cell activity were evaluated (**Figures 5A–C**), as well as the population and activity state of T cells (**Figures 5D, E**).

Regarding the scenario presented by the B cell-mediated response, we observed that titrations appeared modestly after the first dose (**Figure 5A**). In the second immunization, IgG and IgM increased, while IgG1 showed a slight increase, and IgG2 remained low. This scenario continued after the third dose, declining over time. In **Figure 5B**, we observed an increase in memory B cells from the first dose, remaining high after the second and third doses. High levels of IgM were also observed from the second dose, and IgG1 and IgG2 maintained a similar scenario to the previous one. The first dose revealed a peak of B cells presenting antigens, which decreased slightly with the second and third doses. The same scenario was observed in the populations of anergic and antigen-internalizing cells. The first immunization seems to be important for the generation of active B cells that persisted over time after the second and third doses (**Figure 5C**).

Simulations involving the T cell-mediated response demonstrated that the first and second doses generated a high level of memory T cells, and the third dose was crucial for the prevalence of this cell group to persist until the end (**Figure 5D**). The first dose caused active T cells to begin to increase and this profile would be maintained after the administration of the second and third doses. However, this population of active cells would not be sustained until the end. Furthermore, as observed for B cells, the population of anergic T cells seems to remain low (**Figure 5E**).

## 4 Discussion

Recent breakthroughs and progress have been made in exploring vaccine candidates against *P. vivax* malaria. This research tackles three key challenges: 1) Finding better ways to assess vaccine potential before expensive clinical trials, which is due to *P. vivax’s* inability to grow long-term in the lab makes traditional methods even more difficult; 2) Identifying new vaccine targets/novel proteins that could trigger a protective immune response and; 3) improving existing vaccine candidates to maximize their effectiveness. In this scenario, we recently designed a chimeric protein called PvRMC-1, combining conserved epitopes of three known *P. vivax* proteins (PvCyRPA, PvCelTOS, and Pvs25) that were recognized by the immune system of naturally exposed individuals (13). Given that the epitope selection, construct design, recombinant expression, and structural identity were conducted in this previous work, here we aimed to evaluate the immunogenicity of PvRMC-1 formulated in different adjuvants using non-humanized murine models, complementing and validating this chimeric protein as a potential vaccine candidate.

Animal models represent a great basis for vaccine development (30, 31) and adjuvants are also important allies to modulate an immune response strong enough to protect (32, 33). Therefore, we explored three different adjuvants, thus evaluating immunization and recognition of PvRMC-1. The AddaVax adjuvant, characterized as a squalene-based oil-in-water nano-emulsion, is formulated to mimic the Novartis MF59(®) adjuvant, with the activity of eliciting both a Th1 and Th2 response and promoting a faster antibody development in a study involving a malaria Transmission-Blocking Nanovaccine (34). Studies also describe an expanded response of IgG subtypes (35) and activity of neutralizing sporozoites (36); The Stimune® adjuvant, also known as Specol, is an oil-based alternative to Freund’s adjuvant (37), with high efficacy in promoting a strong and persistent antibody level (38, 39), which is a powerful trigger of significant side effects (40) and is frequently employed in veterinary medicine (41, 42); Al(OH)3, the most commonly used chemical as adjuvant (43), including in malaria studies (44, 45), capable to increase the avidity of antibody responses (46–48), performing a higher neutralizing potency of the antibodies (49). A study also found a greater inhibition of PvDBP-II binding to erythrocytes (50) and observed a robust immune response in Aotus spp (51).

Our first data indicate that PVRMC-1 was strongly immunogenic, with high antibody titers induced in all formulations with the different adjuvants used, only after the three-dose schedule. Multiple immunizations expose the immune system to the antigen multiple times, promoting a stronger and longer-lasting immune response. Moreover, the affinity maturation, can selects and proliferates B cells with higher affinity for the specific epitopes from our chimeric antigen. Collectively, although all mouse IgG subclasses recognize a similar set of our antigens, due to the limited serum quantity obtained in experiments and the non-specific inhibitory effect of mouse sera and purified IgG on *P. vivax* asexual stage development *in vitro*, it is not possible to affinity purify the PvRMC-1 specific antibodies for testing their inhibitory potential in invasion experiments at this time. Therefore, special protocols must be established for conducting *in vitro P. vivax* invasion inhibition assays. We highlight Stimune, which presented high antibody titers as a characteristic right after the first immunization. The group vaccinated with AddaVax also showed significant induction of high antibody levels after the initial booster (1:100,000). Interestingly, although immunogenic, the formulations with PBS and Al(OH)3 showed similar results regarding antibody dynamics across the points studied and also in maximum titers. Compared with other findings in the malaria literature, MF59/AddaVax performed lower IgG titers with approximately 10x10^2^ for PfMSP antigen (52), when compared with our data. Another finding is closer to our results, presenting approximately a 10x10^4^ for PvDBP antigen (50). Regarding Al(OH)3, this adjuvant performed lower immunogenicity in a study that related a similar response when compared to our findings, varying between 10x10^4^ and 10x10^5^ for the transmission-blocking candidate Pfs230d1 (44). These findings suggest that our antigen in current formulations can be a potent inductor of the humoral immune response, especially when we observe the performance of the group immunized only with PvRMC-1. Interestingly, in a recent study involving PfCyRPA, AddaVax resulted in a superior immunogenic response when compared to the response with Alhydrogel (which is based in Al(OH)3), but comparable to the potent response achieved with Freund’s formulation and lower than Alhydrogel in the D70, a similar scenario in our study. We observed that the IgG titers between D62 and D83 for AddaVax were slightly lower than the results of these groups (~10^6^ and 10^7^), but the Al(OH)3 titers were similar to those of Alhydrogel (53).

Regarding the IgG subclass evaluation, IgG1, IgG2a, and IgG2b were predominant subclass isotypes in the studied groups. It aligns with previous research (54, 55), though it’s not strongly linked to protection due to its limited ability to activate complement (56, 57). Among IgG subclasses in rodent models, IgG2a and IgG2b are considered the most protective and potent activators of complement and recruitment of relevant Fc-receptors for IgG (FcγRs) (58). This parallel underscore the pivotal role of cytophilic antibodies, particularly IgG1 and IgG3, with similar functional properties in human malaria immunity. The mice IgG3 plays a more modest role compared to the other subtypes, and we also identified the lowest mean OD values, as expected (35). These findings are by the order of prevalence of IgG isotypes observed in a study using AddaVax as an adjuvant, with a prevalence of IgG1 followed by IgG2a, 2b, and IgG3 (50). Despite IgG1 prevalence, IgG2a and IgG2b levels in our study may represent a positive aspect of PvRMC-1-induced response. Moreover, exploring the action of each tested adjuvant, an analysis was performed within each of the tested groups for subclasses, individually compared to the control group (PBS alone). Thus, we observed a pronounced response to Stimune for all subclasses throughout Days 19 and 62. AddaVax also showed a statistically significant response for IgG1 and IgG2a over the two days of testing. The Al(OH)3 adjuvant has been reported as less immunogenic, especially when compared with AddaVax and Stimune (53), which supports our findings in this context. After all vaccine protocols, within the group, tested for IgG2a, a statistical difference was observed compared to the control (isolated PBS) in the Addvax and Stimune groups tested for these subclasses. Given the importance of this subclass (58), we suggest that our protein once again induced a substantial B-cell response. Lastly, although all mouse IgG subclasses recognize a similar set of our antigens, due to the limited serum quantity obtained in experiments and the non-specific inhibitory effect of mouse sera and purified IgG on *P. vivax* asexual stage development *in vitro*, it is not possible to affinity purify the PvRMC-1 specific antibodies for testing their inhibitory potential in invasion experiments at this time. Therefore, special protocols must be established for conducting *in vitro P. vivax* invasion inhibition assays.

In the last investigation about the humoral immune response, aiming to validate the B-cell epitopes in our chimeric protein, we performed a test to confirm the IgG immune response directed towards all epitopes of PvRMC-1 (13). Our results showed effective recognition of the B cell peptides PvCelTOS, PvCyRPA, and Pvs25. These findings corroborate the validation of the structure, suggesting that the B-cell epitopes remained stable and exposed to antibodies even in the Al(OH)3 immunized group, which studies have demonstrated the potential effects of adsorption to aluminum salt adjuvants on the structure and stability of protein antigens (59, 60). We also identified a statistical predominance of the PvCelTOS pool and Pvs25 pools over the PvCyRPA pool in the Stimune group. This was consistent with a lower Reactivity Index observed for PvCyRPA, aligning with literature that indicates this protein is less immunogenic compared to other erythrocytic candidates (24, 61, 62).

In the context of investigating the component peptides of the chimeric structure, we also examined T-cell epitopes from PvCelTOS and PvCyRPA, which were previously predicted and studied in our earlier research (13). Assessing the immune response mediated by T-cells is crucial in the field of vaccinology, as they have a crucial role in adaptive immunity, working in conjunction with B cells and promoting long-lasting immunity. By exploring this response, we can speculate the activation and expansion of helper CD4+ T cells and cytotoxic CD8+ T-cells (63) by IFN-γ production. Moreover, IFN-γ can be produced by CD4+ and CD8+ cells, NK cells (64), it is associated with macrophage activation and antigen presentation, orchestrates the innate immune system, and regulates Th1/Th2 balance (65, 66). This cytokine is also related to the stimulation of IgG2a production (67), activation of cytotoxic cells that play an important role in eliminating cells infected with intracellular pathogens, and the induction of an inflammatory response (64). Moreover, a Th1 ambient seems to be related to isotype switching, resulting in the production of the protective IgG2a and possibly IgG3, exhibiting antibody protective activities that could potentially serve as a broad protective mechanism against bloodstream infections caused by murine malaria parasites (68). Our results indicate that the Stimune group exhibited the highest number of spots, followed by Al(OH)3. In this context, Stimune was the most potent activator of the cellular immune response, as we previously observed in antibody kinetics. Similar findings were observed in other studies with Freund’s adjuvant, a well-known high inductor of IFN-γ (53). The production of IFN-γ is one of the roles associated with AddaVax (36, 50, 69); however, in this study, this adjuvant was not capable of inducing a robust T-cell response. The evident response observed with Al(OH)3 in this study is possibly associated with being a good cytokine inductor, including IFN-γ (53, 70), and the efficacy in adsorbing antigens or immune potentiators through electrostatic attraction, promoting an antigen depot effect (71, 72). One limitation of our study was the lack of exploration of other Th2-associated cytokines. However, given the previously mentioned robust B cell-mediated response, we can speculate that there is also a Th2-mediated response involved. Previous works already demonstrated an association between Stimune and Th2-response in animal models (41, 73) and Al(OH)3 triggering a mixed cytokine profile of type 1/type 2 (73) and related to being a better inductor of cytokines such as IL-4, IL-6, and TNF-α than AddaVax (53).

Regarding the specific T-cell epitope response, PvCelTOS peptide pools exhibited the most significant response, which is also evident when studying the raw number of spots generated, as Pool 2 showed a higher response compared to the others. A possible reason may be that these T-cell epitopes presented a good mean binding prediction score of TCD8+ epitopes IEDB MHC-I (Balb/c mice) and exhibited a high frequency of HLA binding among the 27 HLAs evaluated in a previous study conducted by our group (15).

Lastly, to complement our work, we simulated the action of this protein in potential human models using an Artificial Intelligence tool. Vaccine simulation allows for a faster, more precise, and personalized approach to the development and implementation of vaccines, contributing to accelerated discovery, cost reduction, and improved effectiveness and safety of vaccines (74). When comparing the IgG titers achieved by the antigen, we observe that the administration of all vaccine doses in humans suggests a higher titer than what was observed with our antigen formulated without adjuvant, reaching 7x10^5^. When compared to the end of our kinetic study (Day 136), we see that the titers become equal (1x10^5^). These observations suggest that the antigen alone would be promising in humans and would be capable of generating memory antibodies. Furthermore, the antigen appears to be capable of inducing a good IgG1 response and a lower IgG2 response, which can be interesting since cytophilic antibodies play an important role in human malaria (75, 76). Memory-effector T cells also appear to be increasingly stimulated as the vaccine doses are administered, which suggests a favorable scenario when considering vaccination, as these cells assist in B cell activity and also generate a regulatory response in chronic diseases like malaria (77). However, these cells do not seem to maintain their active state until the end of the follow-up, suggesting that the administration of adjuvants could not only improve this cellular scenario but also maintain IgG titers for a longer duration, resulting in a more potent and therefore longer-lasting and comprehensive response, as observed in our murine models. Further studies will be necessary to validate the different adjuvants in humans, which would constitute another important step in the development of a malaria vaccine. In this context and according to our findings in this work, the mixing of adjuvants has been applied in some malaria studies (78, 79) and the mixture of AddaVax with Al(OH)3 adjuvants could be a good strategy for promoting B-cell response and improve T-cell response, as explored before (80).

In summary, the chimeric recombinant protein PvRMC-1, designed to optimize the immune response against epitopes from three different vaccine candidates was able to induce broad humoral and cellular responses in four different formulations in BALB/c mice. The induced antibodies successfully recognized the recombinant chimeric PvRMC-1 as well as the linear B-cell epitopes of PvCyRPA, PvCelTOS, and Pvs25 individually. Moreover, splenocytes from immunized mice were also able to be activated and produced IFN-γ after the stimulation with peptide epitopes from PvCelTOS and PvCyRPA (pre-erythrocytic and erythrocytic antigens, respectively), confirming the presence of T-cell epitopes in the chimeric antigen. Although the aluminum hydroxide formulation showed important results in the cellular response, Stimune and Addavax formulations were able to generate a more complete response, involving both cellular and humoral responses. Thus, our findings indicate that PvRMC-1 showed promising results and that it’s potential as a multistage vaccine candidate against *P. vivax* can be further defined with preclinical studies in non-human primates and the association of the immune response generated with protection.

## Data availability statement

The raw data supporting the conclusions of this article will be made available by the authors, without undue reservation.

## Ethics statement

study involving BALB/c mice was by the animal welfare protocols established by Bio-Manguinhos (CEUA: LW-13/16). The study was conducted in accordance with the local legislation and institutional requirements.

## Author contributions

AM: Formal analysis, Investigation, Methodology, Validation, Writing – original draft, Writing – review & editing. IS: Writing – review & editing, Methodology. RR: Conceptualization, Writing – review & editing. CR: Writing – review & editing, Methodology. LA: Methodology, Writing – review & editing, Formal analysis. RD: Methodology, Writing – review & editing, Formal analysis. CL-C: Writing – review & editing, Resources. AA: Resources, Writing – review & editing. PN: Resources, Writing – review & editing. FC: Resources, Writing – review & editing. LP: Writing – review & editing, Investigation, Resources. CD-R: Funding acquisition, Writing – review & editing, Investigation, Resources. PT: Writing – review & editing, Formal analysis, Investigation, Resources, Writing – original draft. JL-J: Conceptualization, Formal analysis, Funding acquisition, Investigation, Methodology, Project administration, Resources, Supervision, Validation, Writing – review & editing.
